# Moral distress, resilience and human spirituality in the veterinary clinic

**DOI:** 10.3389/fvets.2026.1922787

**Published:** 2026-09-21

**Authors:** Emil Børty Nielsen, Eva Maria Meier Carlsen, Maja Louise Arendt, Per Torp Sangild

**Affiliations:** 1 Centre for Science and Faith, Faculty of Theology, University of Copenhagen, Copenhagen, Denmark; 2 Department of Veterinary and Animal Sciences, Biomedicine, Faculty of Health Sciences, University of Copenhagen, Copenhagen, Denmark; 3 Department of Clinical Veterinary Sciences, Faculty of Health and Medical Sciences, University of Copenhagen, Copenhagen, Denmark

**Keywords:** animal euthanasia, existential health, holistic health, moral dilemmas, moral distress, moral resilience, science and faith, spiritual health

## Abstract

The spiritual health of veterinarians is challenged by moral distress that expresses a conflict between personal core values and clinical practice. The morally resilient veterinarian experiences and addresses moral distress in constructive ways that express care for patients, owners, colleagues and society. As such, moral resilience is a positive aspect of spiritual health that can be built through reflections and dialogue on the ethical, existential, spiritual and religious questions raised by veterinary practice. Veterinarians often face complex moral dilemmas when attempting to balance the interests of animal patients, their owners, the veterinary business, surrounding society, and themselves. When it is not possible to accommodate all these responsibilities, moral distress may occur, a state associated with compassion fatigue, burnout and depression. Human spirituality concerns a person’s central relationships with the self, others, nature, animals and “the sacred” giving meaning, purpose and values to life. Consequently, moral distress affects not only physical, social and mental health, but also spiritual-existential health and well-being, as it disrupts these core relationships in a way that defies simple technical and practical solutions. We argue that strategies to increase moral resilience (and thus spiritual health) should include conversations with peers and mentors aiming to articulate experiences of moral distress and reinterpret their meaning. Since moral distress in a veterinary clinic involves emotions, values and ideas about the human-animal relationship, resilience-building reflections and conversations must address existential themes like personal responsibility, guilt, acceptance, forgiveness and moral agency. These questions cannot be answered by scientific knowledge alone, but involve personal faith perspectives (secular-existential, spiritual or religious). Consequently, veterinarians, both individually and collectively, should increase attention to the broader existential context of clinical work in order to cultivate moral resilience and enhance spiritual health.

## Introduction

Euthanasia of companion animals in veterinary clinics can be an emotionally ambivalent experience not only for owners, but also for veterinary medical and nursing staff and students in training (1). Taking the life of a sentient being, showing an instinctive will to live and a deep relation with humans, is a moral act at the intersection between life and death. As such, it carries existential, ethical and even spiritual dimensions that require careful deliberation. In some situations, euthanasia may be experienced as an act of compassion that relieves an animal from suffering; in other situations, it may be a cause of moral concerns and emotional distress.

Moral distress refers to a stressful and challenging emotional state that arises when care professionals are unable to act according to their personal or professional moral values (2). This emotional state may occur when veterinarians face conflicting views of the moral status of animals, disagreements over how resources should be allocated, or pressures from societal and institutional structures. Living in alignment with one’s core values is, as we argue below, central to human spiritual health and directly or indirectly influences physical, mental and social health (Figure 1).

**Figure 1:**
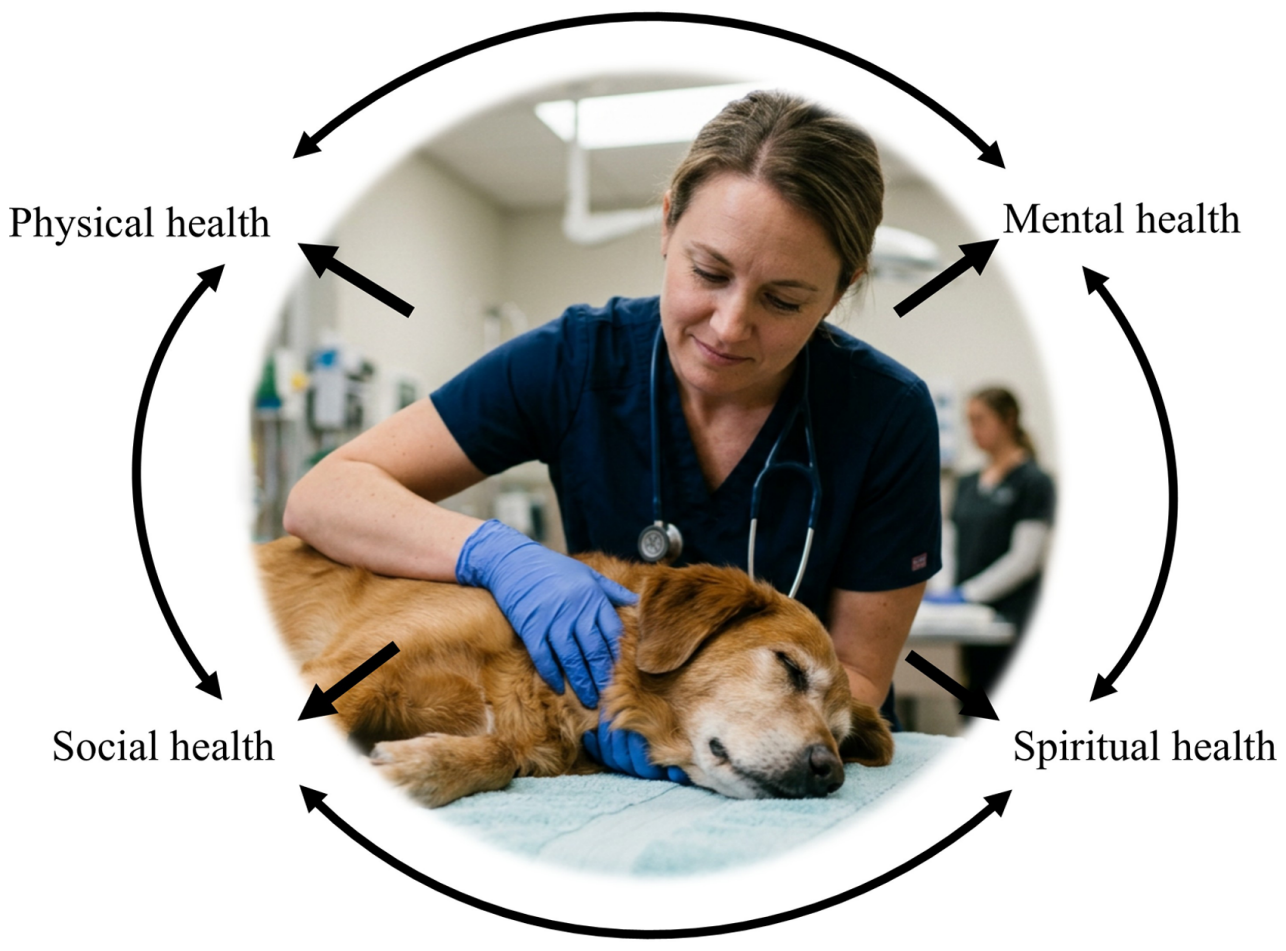
The four interacting domains of human health (physical, mental, social, spiritual) used in palliative care medicine (8) are also relevant in veterinary clinics. Moral distress in veterinary work may relate to all domains of human health, including spiritual health as it is a disruption of personal meaning, purpose, and identity with loss of the ability to live in alignment with own core values. Personal reflection and communal conversations with peers and mentors on topics like responsibility guilt, acceptance, forgiveness, and agency may be an approach to develop moral resilience, an important aspect of mental and spiritual health. (Photo generated by Perplexity AI).

Moral distress is a natural, socially important emotional response to perceived moral injustice. As such, it can be compared to our ability to feel physical pain. Even though pain is unwanted, it plays an important function in keeping us safe. Without moral distress, we could probably not form functioning societies where humans can flourish. Moral resilience is thus not about removing our capability for moral distress but experiencing moral distress in constructive proportions and learning to handle it in constructive ways.

Moral distress is a result of how we morally interpret a situation both cognitively and emotionally. The beliefs and convictions engaged in such interpretation can be misguided and thus wrongly elicit or fail to elicit moral distress as a reaction to a particular situation. An important aspect in handling moral distress and developing moral resilience is thus to help veterinarians reflect on and discuss how they interpret the moral context of clinical work with focus on moral dilemmas and situations eliciting moral distress (like euthanasia). Science plays an important role in qualifying veterinarians’ understanding of clinical situations. Some aspects, though, defy scientific knowledge and can probably never be settled scientifically (e.g., questions of animal worth, rights and experiences). Consequently, veterinary work inevitably involves personal faith.

This article is not about normative animal ethics (3–5) but about how veterinarians can handle the moral and existential uncertainties inherent to clinical work. Thus, it is about the spiritual health and well-being of veterinarians. It is important to note, however, that such normative theories and beliefs are an important part of the reflection and reinterpretation that we suggest may aid moral resilience. Both normative ethics and veterinary health, though, justify that researchers and practitioners take moral distress seriously and work to change problematic cultures. Though the focus of the article is on veterinarians, the main argument is equally relevant for other groups of professionals working with production animals, wild animals, zoo animals or experimental animals (see Figure 2).

**Figure 2:**
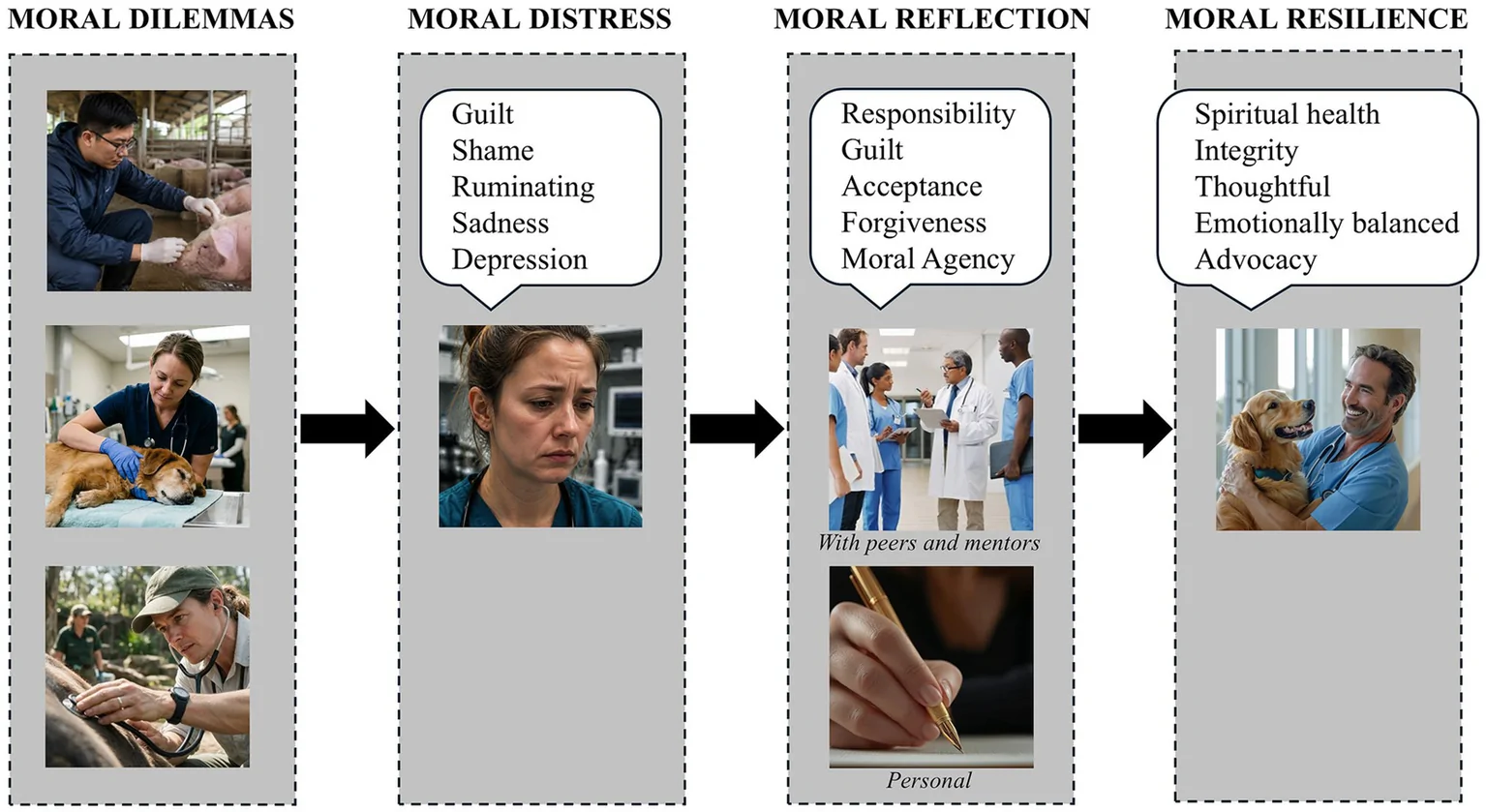
An approach to identifying and handling moral distress in veterinary work through personal reflection and communal conversations with peers and mentors to improve spiritual-existential health and moral resilience. (Images by Tyli Jura and Maria Moczydlak on Pixabay and AI-generated using Perplexity and ChatGPT).

From studies among human health care workers, moral distress is known to affect the physical, mental and social health of care professionals (i.e., the complete human health according to the WHO constitution from 1946). However, the nature of moral distress reaches beyond this three-dimensional model of health and disrupts what in some health contexts, especially palliative care medicine, has been termed “spiritual health” (6–8). On a superficial level the model can be understood as describing four isolated domains of human health. For example, patients need not only medical doctors but also psychologists, social workers and hospital chaplains to treat different isolated problems in their holistic health. On a deeper level, though, the four aspects are present in all medical conditions and interact on various levels. For example, a broken leg is a physical ailment requiring physical treatment. At the same time, it may have social aspects (the patient cannot go to school), psychological aspects (the patient feels depressed from staying in bed) and existential-spiritual aspects (the patient feels they cannot engage in their dream of becoming a professional soccer player) (Figure 1). Some researchers prefer to name this fourth domain of human health “existential health” to avoid religious connotations and alienating non-religious care professionals and patients (9).

In this article, we follow an internationally recognized consensus definition of human spirituality from palliative care medicine as “A dynamic and intrinsic aspect of humanity through which persons seek ultimate meaning, purpose, and transcendence, and experience relationships to self, family, others, community, society, nature, and the significant or sacred. Spirituality is expressed through beliefs, values, traditions, and practices*”* (8). More brief, spirituality is the search, experience and expression of what makes life meaningful. Here, the relationships that give life meaning and purpose may include family, work, sport, religion, political commitments, nature and animals (10). Human spirituality is related to personal or communal faith convictions (secular-existential, spiritual or religious) since these aspects of human life go beyond scientific knowledge (11, 12). A person’s core values are thus aspect of personal faith that expresses what relationships are important and meaningful to them. Failing to act in accordance with one’s core values is thus a reduction of one’s spiritual health and may elicit moral distress.

In drawing attention to faith and spirituality in personal wellbeing, we do not wish to exclude or reject other relevant academic and practical approaches to understand and handle moral distress among veterinarians. Such other approaches may relate to their basic physiological needs, psychological wellbeing, ethical reflection, social support or organizational structures (13, 14). However, these other factors may interact with personal faith and spirituality to support overall moral resilience, as suggested in Figure 1. Attention to the existential aspects of personal wellbeing, in addition to more classical occupational health approaches, is difficult in many professional contexts because faith and spirituality go beyond traditional analytical knowledge and are based more on subjective experience, ultimate concerns, hope, trust and worldview assumptions than on objective knowledge (11, 12). Yet, we argue they are equally important in a holistic approach to human health at both human hospitals and in veterinary clinics. Spiritual veterinary care, with professional chaplains, is now present in some veterinary clinics and universities (15, 16) (cf. https://aavchap.org/; https://chaplaincy.tufts.edu). Thus, the existential dilemmas for human and veterinary hospital personnel, caretakers and family following a seriously ill or dying patient, share many similarities.

Care professionals handle moral distress in various ways. Some live with the moral distress engendered by the profession. This is an unsustainable solution because of the negative health outcomes associated with being in a state of consistent and severe moral distress. Other care professionals become indifferent to the moral dilemmas of veterinary practice, thus removing their discomfort. This, however, is also unsustainable because morally insensitive veterinarians might not react to, for example, mistreatment of animals or important needs of the owners. To avoid both these deficient solutions, we point to a third option: Moral resilience. Based on inspiration from nursing, psychology, philosophy and theology, we draw the contours of a path that preserve moral awareness, integrity, and agency while avoiding the debilitating effects of moral distress. Moral resilience can thus be viewed as an aspect of sound spiritual health where the veterinarian is able to stay true to their core values in a complex world of uncertainty, moral dilemmas and unanswered questions.

This article has four parts. First, we present some empirical studies to demonstrate that moral distress is indeed a problem in veterinary clinical practice. Second, we present the concept of moral resilience as an aspect of spiritual health that helps veterinarians handle moral dilemmas and distress. Third, we focus on cognitive reinterpretation of morally stressful events as a central aspect of building moral resilience. We propose that such reinterpretation may be supported through peer conversations, journaling and mentorship. Last, we venture into the content of reinterpretation and explore guilt, responsibility, acceptance, forgiveness and agency. These are overlapping psychological, philosophical, ethical and theological concepts that may help us understand and handle moral distress from the perspective of the individual person.

Our aim with the last section is not to settle these normative questions, but to stimulate personal reflection and reflective conversations among veterinary students and professionals. Science cannot definitively answer how we should act in our relationship with animals. Comprehension and decisions should be based on scientific knowledge, but inevitably, personal values, convictions, intuitions and experiences also play a role. Each veterinarian must find their own answers to the existential questions related to veterinary work, including animal euthanasia. This does not mean, though, that individual veterinarians should tackle complex moral and existential questions alone. Veterinarians can learn from each other’s experiences and personal reflections, and personal faith commitments may (unknowingly) be shared by colleagues. Yet, it is important to embrace a range of attitudes and a work culture where the individual veterinarian has freedom to find their own path inspired by both hard scientific knowledge and softer personal experiences, commitments and worldview assumptions.

In drawing attention to existential issues (not only ethical issues), we follow a growing trend in the field of veterinary science to build academic bridges to the humanities. Veterinary humanities, with its inspiration from medical humanities, is a growing field still in its infancy (1, 17). Considering the complexity of the human-animal relationship (physical, social, mental, spiritual), veterinary humanities can draw attention to moral distress, resilience and the role of faith, spirituality and existential uncertainty in a job that needs to care for the health of both animals and humans.

### Moral distress among veterinarians

In the health sector, moral distress was first described among hospital nurses. It was defined by ethicist Andrew Jameton in 1984 as “the experience of knowing the right thing to do while being in a situation in which it is nearly impossible to do it” (2). During the 1990ies the concept was applied to a range of other occupations like soldiers, police officers, journalists, veterinarians, animal shelter workers and health care workers in general. Moral distress is experienced when a person’s deeply held moral values are transgressed, either as a perpetrator or witness (18) and can be defined in the following way:

“Distress that arises because personal experience disrupts or threatens: (a) one’s sense of the goodness of oneself, of others, of institutions, or of what are understood to be higher powers, or (b) one’s beliefs or intuitions about right and wrong, or good and evil”

(19).

In this article, we will focus on the moral distress arising from being a perpetrator not acting in accordance with one’s moral beliefs. We note though that moral distress can also arise from being a witness to moral transgression. In this sense, the clinic functions as a moral community where the moral choices of one veterinarian may influence the spiritual health of their colleagues.

Moral distress is a situational emotion that may leave behind a “moral residue” impacting the continued life of the veterinarian. Such residue might lead to gradual moral deterioration, and even injury where the veterinarian becomes detached from work and stops caring about the welfare of patients and clients. Not only the intensity, but also the persistence of moral distress experienced may exert a lasting impact on the health of veterinarians resulting in moral injury or moral injury disorder (19, 20).

Moral distress has been shown to have a long list of negative consequences among health professionals. These include anxiety, guilt, depression, low job satisfaction, early retirement, post-traumatic stress, treatment errors, depersonalized care, and negative career identity (21). Among veterinarians, moral distress may cause stress, burnout, compassion fatigue and other mental health problems (21, 22) influencing all aspects of human health, and in the worst cases leading to suicide (23). The stressors themselves and their health effects are highly culture- and context-dependent and could be more intense in large, urban communities where meeting clients´ needs and expectations could be more difficult than in rural communities. Studies in Hong Kong showed that veterinary stress extended far beyond the health of the animal patients but was much dependent on adequate and satisfying communication with clients and surrounding society (23, 24).

Veterinarians are often faced with difficult decisions that involve life or death for their patient (25). In a clinical context many different situations may engender moral distress like euthanasia, futile treatment, inadequate informed consent, deception, false hope, errors, elimination of lifesaving treatment, and violating patient rights or autonomy (21). For instance, moral distress can be experienced in situations where a client cannot or will not pay for treatment of a suffering animal. Other cases include convenience euthanasia or a client not allowing a suffering animal to be euthanized because of the importance of the animal for the owner (26). Making wrong treatment decisions or failing to diagnose a condition may also contribute to moral distress and self-blame due to the negative consequences for patient and owner.

Empirical studies indicate that moral distress is a problem in the veterinary field, like in many other health care domains. In one study, the majority of UK veterinarians experienced morally injurious events that were associated with symptoms of PTSD (27). In another study (28), the majority of US veterinarians experienced that animal owners’ financial limitations compromised patient care, potentially leading to symptoms of moral distress. A survey of US veterinarians (22) showed that the majority of these experienced moderate or severe distress when they could not “do the right thing” and provide the care they thought was appropriate.

In Australia, the majority of veterinary students in a survey thought that their primary focus as veterinarians should be to care for the interests of all animals (29). Kipperman et al. found that among US veterinarians only half of them described themselves as prioritizing patient interest (28). This indicates a conflict between student expectations and occupational reality, which could be a cause of increased moral distress during their first years of clinical work. For veterinary professionals, moral conflicts were a common reason for leaving the profession (30).

Empirical research on moral distress among veterinarians is still limited and more research is needed to determine the scope of the problem (21, 31). Still, existing evidence suggests that moral distress is a prevalent problem with potential negative consequences for both veterinarians and their patients. To understand the problem of moral distress and design interventions, we suggest that theoretical perspectives from psychology, philosophy, ethics and theology are needed and can complement existing quantitative and qualitative studies.

### Moral resilience in human health care settings

The traditional way of training veterinarians and students to engage moral dilemmas is by improving their education in formal ethics. Such programs may have some effect, but will not solve the problem of moral distress alone (32). One study found that among veterinarians in veterinary clinics the most used method for deliberating moral dilemma was “gut feeling” based on personal values followed by talking to colleagues or friends. Recalling ethical theories was much less used (28). It is difficult to say precisely what “gut feeling” is. It may be interpreted as an affective response to the situation (rather than cognitive), guiding practical actions. Such affective response may be grounded in the veterinarians’ core values, experiences and personal faith (whether religious or not). Since formal ethics is primarily cognitive (rather than affective), its effect on “gut feeling” is likely to be limited. A third way should thus take a more holistic approach to how humans navigate moral distress going beyond classical training in formal ethics. The concept of “moral resilience” developed in the nursing field is an attempt to formulate a more holistic approach to engage moral distress that can be transferred to the veterinary field.

Moral resilience is defined as “the capacity of an individual to sustain or restore their integrity in response to moral complexity, confusion, distress, or setbacks” (33, 34). Moral resilience has five aspects: [1] integrity (a state of self-apprehension where core values and actions are seen as aligned), [2] buoyancy (an ability to recover integrity after loss of integrity), [3] moral efficacy (an ability to see how one can act in alignment with one’s values despite perceived moral problems or dilemmas), [4] self-regulation (an ability to monitor one’s emotions, e.g., anger, sadness, moral distress, and not necessarily act according to them) and [5] self-stewardship (an ability to take care of oneself and one’s basic needs). As such, moral resilience is an affective-moral state of self-apprehension [1] that can be recovered and maintained with at least four abilities [2–5] (see Figure 3).

**Figure 3:**
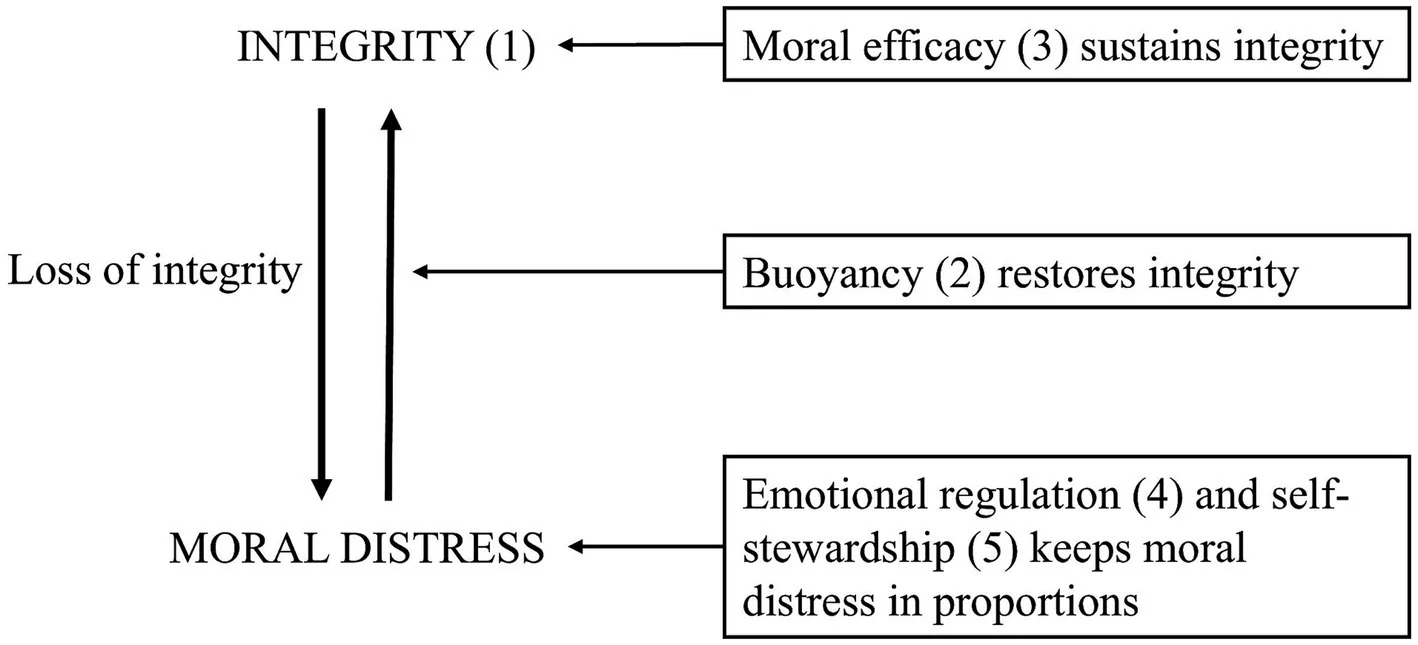
The five aspects of moral resilience.

The central concept of moral resilience is *integrity*. Integrity is a personal state of harmony in which one’s inner values are aligned with one’s choices and actions. It is thus a state in which veterinarians can adequately adhere to their core values and faith commitments. When a person transgresses moral values, integrity is disrupted. This loss of moral integrity is experienced as moral distress (35).

Morally resilient people are *buoyant*. This means that after loss of integrity they find ways to recover it. This can be done through recalling one’s personal commitments or through a personal reorientation that leads to personal growth. Buoyancy involves being able to identify the feeling of moral distress as moral distress (35). Such affect labeling can regulate emotions and thereby help veterinarians understand what is experienced, as well as reducing distress (36). Buoyancy involves the ability to reinterpret the meaning of the event that caused moral distress. Reflecting on a situation experienced as morally stressful, a veterinarian might, for example, acknowledge that what happened with the animal was not their personal responsibility. Despite a wrong decision for an animal patient, the veterinarian tried their best, with the information available at the time and remained faithful to core values. Reinterpreting the meaning of an event that caused moral distress is thus an important part of moral repair and counters moral deterioration over time (35).

The third ability is *moral efficacy*. Resilient veterinarians believe they are able to advocate on behalf of patients (and owners) and challenge what they perceive as morally problematic situations and structures. Such advocacy, even when it fails, helps maintain a sense of personal moral integrity. After all, the veterinarian did what they could on behalf of their patient. As such moral efficacy helps sustain personal integrity. The fourth ability is *self-regulation*. To self-regulate, the veterinarian must be conscious of their own emotions and avoid being swept away by them. Veterinarians must recognize their own feelings, judge why specific situations awaken these feelings and respond constructively grounded in their own inner values. Rushton advises nurses to practice mindfulness as a tool to help them be attentive to their own emotions and learn to ground their decision in their core values rather than being affected too much by emotions of morally difficult situations (35).

Lastly, the resilient veterinarian must assert *self-stewardship*. If basic needs for food, sleep, security and social company are not met, one is more likely to make medical mistakes that could cause moral distress. Further, a self-neglected, stressed or lonely person may experience moral distress out of proportions, when facing ethical dilemmas, or try to solve dilemmas in inappropriate ways. These latter two skills help the veterinarian experience moral distress in constructive proportions. It is not in the interest of patients or their owners that veterinarians are overwhelmed by debilitating moral distress, leading to a deterioration of personal identity. Rather, moral distress should call for self-reflection and actions that feed compassion and moral efficacy (35).

We believe the concept of moral resilience is helpful in devising interventions that can help veterinarians and clinics handle moral distress. One caveat should be noted though. The concept of moral resilience risks individualizing the problem. Moral distress is not solely (or primarily) a responsibility of the individual veterinarian. Veterinary clinics and companies are responsible for creating work environments that handle moral distress, foster moral resilience and work toward a culture of ethical care for animals, veterinarians and owners. Further, society at large, politicians and citizens, are partly responsible for a system that often mistreats animals. Consequently, the moral issues that give rise to moral distress among veterinarians must be dealt with at all societal levels, not only as an individual problem.

The concept of moral resilience opens several venues for exploration and application in a clinical setting. Moral distress must be addressed at all levels of human need from the basic, needs for food, sleep and security to the higher needs of self-esteem, self-actualization and spirituality (13, 37). In the following, we will focus on the concept of cognitive reinterpretation. With this choice, we do not wish to deny the importance of basic needs like food, sleep, breaks, friendship and emotional safety for constructively experiencing and handling moral distress. Similarly, we also recognize that experience-based practices less dependent on cognition and language like art, music, literature, mindfulness, nature walks, meditation, prayer and rituals could hold potential for handling moral distress and building moral resilience at both personal and communal levels. This would align with the hypothesis that both human-human and human-animal relationships extend beyond physical, social and mental health effects to include human spirituality (38). Yet, cognitive practices such as conversations with mentors and peers as well as personal reflection also hold potential for building moral resilience, and this is the focus of this article.

As a spiritual-existential problem, moral distress is partly engendered by personal beliefs about moral duties, animal dignity, guilt and forgiveness, as well as by grand narratives about the relationship between humans and animals. Many of these ideas go beyond what science can answer and are thus ultimately a matter of personal faith, even though they are often shared within workplaces, cultures and societies. Resilience-building cognitive reinterpretation not only concerns concrete questions about who did what in a stressful situation but also the large existential-religious questions about the human-animal relation and questions of personal meaning, purpose and hope. Consequently, we believe veterinarians must dare to venture into the difficult terrain of faith in personal reflection and communal conversations.

### Creating space for reflection and conversation

Cognitive reinterpretation (or reappraisal) is a process through which an emotional response is influenced through influencing how one thinks (cognitively) about the situation in question (39). Moral distress may thus be influenced through a reflective attempt to understand a past event from new perspectives, or in light of new ideas. Successful reinterpretation elicits new affective responses (or negates an old response) to the past situation and may influence how similar situations are experienced in the future (thus fostering moral resilience). To reinterpret an event, the veterinarian must tell the story of what happened (verbalization) and express what they thought and felt in the situation. When the situation has been verbalized, the events, values and ideas it contains can be reassessed through reflective writing or conversation open to new perspectives. Even ordinary storytelling among colleagues at the coffee machine may facilitate constructive reinterpretation and thus be important for creating a spiritually healthy work environment. A broad range of structured approaches in the clinic can be conceptualized to facilitate such reflective conversations like clinical rounds, a monthly peer walk-and-talk-session, a dedicated bench for sharing difficult experiences (40) or a formal mentorship program, including scheduled conversations on moral concerns.

Conversations with a colleague, mentor, friend, partner or even a stranger can thus help the veterinarian to connect with stressful experiences, verbalize them and reinterpret their meaning (22, 27). The strategy faces two challenges. First, these practices demand time and attention which can be difficult to prioritize in veterinary practice where strict time schedules apply to daily work. How much time that should be allocated to this process depends on the context. Given the fact that veterinarians often make decisions of life and death, and that empirical evidence point to moral distress as a problem for the physical, mental, social and spiritual health of veterinarians, it seems like a moral prerogative that some time is allocated to address moral dilemmas and moral distress. It may even reflect positively on the bottom-line through reduced turnover and improved job satisfaction, patient care and mental health. The second challenge is that peer-conversations and mentoring require trust and emotional safety. Veterinarians need to feel safe in sharing difficult emotions and uncertainties without being judged. Senior veterinarians have a special responsibility for creating an environment where junior veterinarians feel understood and respected when sharing personal thoughts, emotions and core values.

Conversations can be supplemented by structured journaling relating to stressful events in the clinic. Writing takes time and forces the writer to focus on the ideas written. Empirical research has documented that writing on topics that awakens strong emotions has positive effects on human health, probably because writing recognizes and structures difficult experiences through language (41, 42). Practices similar to journaling are already in place in the form of medical records, referral letters and discharge letters. These help the veterinarian (and owner) understand what has taken place in the clinic and are especially important if the clinical process was acute and chaotic or if the information given at a consult was complex. However, the formal records do not include the veterinarian’s personal emotions, uncertainties and moral judgments. These topics, though, are important for building moral resilience and may be reflected upon in a private journal. Journaling might best be seen as a private initiative outside work to ensure privacy since the topics are very sensitive. Still, it may help the individual veterinarian manage moral distress and qualify peer-conversations in the clinic. Further, journaling as a strategy is deficient due to its individualist nature. Moral concerns must be addressed in public discussion among veterinarians in the clinic and profession. Still, journaling may hold potential for synergizing with conversational approaches through helping the veterinarian to verbalize and reflect and thus to bring more to the shared discussion.

Moral reflection is not in itself a way of removing moral distress. Quite the contrary, moral reflection could elicit moral distress and uncertainty because one starts to question unquestioned assumptions and see the clinical practice from new perspectives. Yet, moral reflection and acknowledged uncertainties are important parts of the process toward moral resilience, spiritual health and a culture of ethical care. Having suggested above some ways in which it is possible to build moral resilience in the clinic through cognitive reinterpretation, we will now turn to the *content* of such reinterpretation, taking inspiration from the psychology, philosophy and theology of moral distress. We will focus on five key topics for reflection: responsibility, guilt, acceptance, forgiveness and agency. The aim is to open a reflective conversation on how veterinarians can navigate moral distress from the perspective of personal faith and spirituality.

### Five central themes in the philosophy of moral distress

#### Responsibility

Responsibility is a prerequisite for having moral duties and being blameworthy. If we have no responsibility in a given situation then nobody can blame us. Responsibility is a non-empirical notion. You can measure qualitatively and quantitatively whether people report that they feel they are responsible. You cannot measure responsibility as a moral notion. Consequently, it is a construct of personal and societal faith, and it is constantly up for cultural discussion who is responsible for what.

There are at least three central ways of ascribing responsibility: Abilities, promises and law. Responsibility is often thought to be proportional with abilities. Parents, for example, are responsible for their small children and not vice versa. Through life the roles of responsibility change and finally flip when children become mature adults and parents become old and frail. Applied to veterinarians in the clinic this means that since they have relevant skills, knowledge and tools they have a special responsibility for animal health and well-being. In the same sense their employer is responsible for organizing a work environment that allows the veterinarian to perform their work responsibly.

Responsibility can also be established through promises and oaths. If you promise someone that you will do something, then you are (in most instances) morally responsible for doing it. In many countries veterinarians enter the profession through taking an oath whereby they take on special responsibilities on behalf of the profession. The World Veterinary Association formulates the veterinarian oath in the following way:

*I, as a Member of the Global Veterinary Profession, do solemnly swear to use my scientific knowledge, skill and training: To prevent, diagnose, treat and manage pain and disease in all animal species to the best of my ability in keeping with the principles of veterinary ethics and relevant law. To communicate and help prevent the impact of zoonotic diseases and public health risks and environmental contaminants of animal origin. To advocate for the humane and sustainable management of terrestrial, aerial, and aquatic animals in their diverse ecosystems through stewardship to reduce environmental impacts. To contribute to animal, human, and environmental health by continually developing my competencies as a health professional, acting with integrity and compassion, and promoting health and welfare of humans and animals* (43).

This oath establishes a special veterinary responsibility for animals and provides guidelines for veterinary practice although it does not solve all questions as to the precise nature and scope of this responsibility. Lastly, public law establishes special rights and responsibilities for veterinarians. These three external aspects of establishing responsibility must be placed in dialogue with the veterinarian’s own sense of responsibility expressing their own core values, existential commitments and spirituality. The scope of the veterinarian’s responsibility cannot be scientifically determined and will therefore always be a topic for individual reflection and public discussion.

The veterinary oath, for example, functions to place the veterinarian under a clear moral responsibility that goes beyond merely following the law, thereby promoting ethical care for their animal patients. At the same time, it can be argued that the oath is too demanding and unrealistic and may thus create moral distress among veterinarians that may counteract ethical care. Veterinarians thus need to develop their own (and shared) interpretations of the oath in light of their specific contexts to find a healthy and sustainable understanding of the scope of their responsibility. At the same time, clinical communities and the veterinary profession must foster a continued conversation on responsibility with special attention to helping new veterinarians enter the conversation.

#### Guilt

The concept of responsibility implies that one can fail one’s responsibility. That is, you can become guilty of having let down someone (e.g., animal, owner, company, society, self, God) toward whom you have a responsibility. This someone is then entitled to blame you for your course of action. The feeling of guilt is a central aspect of moral distress. In the moral resilience framework above, guilt is presented as a “loss of moral integrity”. Here we introduce the concept of guilt because it gives attention to the web of relations of responsibility the veterinarian is a part of, rather than seeing them as isolated individuals.

Feelings of guilt can arise in many situations in clinical work. A veterinarian euthanizing a healthy dog on the owner’s request may feel guilt. Or they may feel guilt toward both animal and owner in situations where the owner cannot pay for medical aid and the animal must be euthanized rather than treated. Do such cases mean that veterinarians really are guilty and blameworthy (from a normative perspective)? A central utilitarian moral principle states that actions that would otherwise be immoral are morally permissible if they serve a sufficiently important purpose (44). Euthanizing a dog is morally justified, if it suffers pain and cannot be treated. Exposing animals to disease in experiments may be morally justified if the experiments are expected to yield important knowledge, for example, for combating severe human diseases. This principle means that even though euthanizing a dog is a bad thing in itself, the morality of the action cannot be determined without knowing its purpose and context. If euthanasia is carried out for a morally good reason, then the veterinarian is innocent (retains their personal integrity) – even if they feel guilty.

The problem, of course, is that it can be hard to determine what counts as a “sufficiently good reason” in each case. This, in part, depends on how one views the moral status of animals (companion, farm or wild animals alike), relative to humans. Veterinarians may thus sometime experience a moral uncertainty where they are not sure whether their actions are justified or not. Consequently, they might feel uncertain as to whether they are doing the right thing or have violated their own moral principles, core values and existential commitments. Such uncertainty alone may sometimes be enough to elicit feelings of guilt and moral distress. Veterinarians must learn to live with such moral uncertainty navigating between the interests of animals, owners, company and society, and their own core values and faith convictions.

#### Acceptance

Acceptance is a key concept in learning to live with the moral dilemmas of the veterinary profession. It involves learning to accept that we are limited creatures living in a broken world. This may mean that both our responsibility and ensuing guilt are sometimes smaller than some realize.

“Ought implies can” is an important moral principle attributed to philosopher Immanuel Kant (45). The principle states that you are only morally responsible for doing X, if it is within your power to do X. Therefore, if a veterinarian cannot help a dog, then they are neither responsible nor blameworthy for not helping the dog. The clinical reality of life as a veterinarian in many situations is that of limited agency. Moral dilemmas and problematic treatment of animals are constantly produced, not necessarily by immoral owners or veterinarians, but by structures in society and nature. Nature has produced animals that need other animals (e.g., carnivorous species need other animals for food). Humans have biologically and culturally evolved to “need” other animals for food, clothes, tools, workforce and company. Further, modern societies have complex economic, juridical, political and cultural structures that make it difficult to improve the welfare, agency and independence of animals although many express that this is urgently needed. The individual veterinarian has no or limited power over such natural and societal structures and is therefore not responsible for the negative consequences they produce. Acceptance of this fact may be an important aspect in coming to terms with moral distress.

This does relieve veterinarians of their special responsibility for animal health and welfare. Veterinarians have, for example, a professional responsibility to unite and challenge unjust and harmful societal structures. The difficult part is assessing what one can reasonably do, and what one cannot as an individual or a veterinary community. Hence, the wisdom of the serenity prayer is relevant to veterinarians facing difficult moral dilemmas, as written by the theologian Reinhold Niebuhr for the US armed forces in 1944:

*God, give us grace.*

*to accept with serenity.*

*the things that cannot be changed,*

*courage to change the things.*

*that should be changed,*

*and the wisdom to distinguish.*

*the one from the other* (46).

It requires wisdom to discern the difference between the responsibility that our power places upon us, and the limitations that we must accept. Since uncertainty about the scope of one’s responsibility may be a cause of moral distress, we suggest that acquiring the wisdom of discernment is a trait of moral resilience and spiritual health. Veterinarians, as all others, need to live with uncertainty both in the form of accepting it as an unavoidable part of life, and in acquiring the wisdom required to navigate in a complex world. Such wisdom is partly built when veterinarians reflect on and discuss moral dilemmas, emotions and ideas with peers. The answer is not black-and-white. Consequently, being able to live in life’s gray zones of uncertainty, both scientifically and existentially, is an important goal in growing moral resilience and spiritual health.

#### Forgiveness

The experience of moral distress not only raises the question of guilt but also of forgiveness. In the face of personal guilt and failure, the veterinarian must regain moral integrity and the belief that they are a good person living in accordance with sound values. Forgiveness can be understood as repair of a relationship damaged through wronging the other (negating guilt). This raises the question of who the veterinarian is relating to, and how the relation can be restored. In the following, we will shortly address five kinds of relationships: Animal patients, owners, God, higher principles and self.

When we wrong another person, we damage our relationship with them. In theory, the same goes for animals. Even though some higher animals have what seems to us to be some rudimentary sense of moral sense, it is a stretch to speak of a damaged human-animal relationship in need of repair, as the animal seemingly has no apparent conception of such a moral relationship. Further, in cases of animal euthanasia, the relationship has ended. Consequently, forgiveness functions differently in relation to animals than in relation to human individuals. The veterinarian may still feel a need for forgiveness in certain situations and since this cannot be sought directly from the wronged, it must be attained indirectly through seeking forgiveness from oneself or from God (e.g., through prayer or ritual).

In wronging another, we also wrong those who are in a close relation to them. The same is true for animals. A medical error also damages the relationship with the owner of the animal. Even though the veterinarian may feel no guilt, especially if they have done their utmost and have followed guidelines, there is still a relationship that needs repair and anger to be managed. Opposite situations may also occur, for example, in situations of convenience euthanasia where the veterinarians feel guilt, but the owners feel none. In such situation, forgiveness can neither be sought from the animal nor the owner, but must be sought in oneself, colleagues or faith in a transcendent power, higher principles, nature or God.

In the Western world, the discourse on guilt and forgiveness has taken place mainly in a Christian theological tradition. Viewed from this tradition, human sin and moral failure damage the relationship to God. Consequently, humans need divine forgiveness which mean restoration of the divine-human relationship. Thus, for Christian veterinarians there is an important religious aspect of moral distress and their religion may hold specific resources for building moral resilience, such as prayer, confession and rituals. The same goes for other religions. For non-religious (secular) veterinarians there may still be a sense in which perceived immoral acts become a violation of core values related to “a higher principle” or “The Universe.” Their moral distress results not primarily from violating commonly agreed norms and laws in human cultures, but something they perceive as being “higher,” something they regard as existentially or spiritually decisive. It seems important that personal reflection and peer conversations on moral distress in veterinary practices have room for such experiences and their interpretation in secular, spiritual and religious worldviews. To build moral resilience and spiritual health, individuals and communities must find their own answers and understanding based on scientific knowledge as well as personal experience, belief and conviction, in dialogue with peers, clients and society.

Many young people seem attracted to the veterinary profession because they feel a strong desire to care for animals (1). When they fail to live in alignment with this core value, they not only fail animals, but also themselves. Consequently, they may need to forgive themselves. Self-forgiveness can be difficult because we cannot fool ourselves. Forgiving oneself often involves authentic regret and remorse. In remorse, moral failures are recognized, core values reaffirmed, and actions are taken to prevent future violations of core values. Remorse is thus the opposite of cynicism. Remorse is choosing to care for the vulnerable despite failure, challenges, sacrifices and unjust conditions in nature as well as in societal structures. Serious self-forgiveness is not a superficial removal of moral distress but include personal attention to both root problems and possible solutions – even when these are multifaceted and viewed differently across individuals, clinics, cultures and societies.

#### Moral agency

Wisdom and experience help the veterinarian determine what is within their scope of personal responsibility. Self-forgiveness contains an impulse for action on behalf of their core values. Both are thus connected to their personal moral agency and their ability to make moral choices to benefit patients, clients and society. Moral agency can take many different forms. Some action may focus on the particular patient and client in front of the veterinarian. When the veterinarian cares for an animal patient or client, they do more than just perform a paid service. As such, it is a moral action and an expression of moral agency. Another kind of moral agency concerns animal advocacy. When, for example, a veterinarian attempts to dissuade convenience euthanasia, or advocate for euthanizing a suffering animal, they also exercise moral agency. It can also take more political forms where the veterinarian advocates for more appropriate care for animals and challenges structures perceived as unjust (14).

In practicing moral agency, the veterinarian acts on their core values in a way that goes beyond what they are paid to do. In this way, veterinarians may express to themselves and others that their work is not merely a paid job but an existential orientation, an expression of love and personal purpose. Such self-apprehension is important for spiritual health and a sense of self-worth. Further, it counterbalances experiences of moral distress implicitly stating that the veterinarian is a bad person who wrongs animals and clients. It also counters a moral deterioration in which veterinarians come to see themselves as powerless tools of unchangeable systems. Even under the most challenging circumstances, we can still find places, no matter how small, where we can choose to act on behalf of what we believe to be true and important. We always have some kind of choice, even under high pressure (47). Asserting moral agency is thus an important element of moral resilience, self-esteem and spiritual health.

## Conclusion

In this article, we have examined moral distress as a spiritual problem in veterinary clinics. Empirical evidence demonstrates that moral distress is a problem in the veterinary profession as in other health care professions. We have presented a framework of moral resilience developed from the nursing field and applied it to the veterinary profession. A central element in moral resilience is the ability to cognitively reinterpret stressful situations seeing them from new perspectives. In the clinic, such reinterpretation can be facilitated through peer-conversations, mentoring and journaling. We suggest that responsibility, guilt, acceptance, forgiveness and moral agency are important topics in personal and communal reflections on moral distress. These topics do not have one clear scientific answer as a basis for a universal guideline to action. Consequently, individuals should be willing to enter into the difficult terrain of personal faith, spirituality and existential uncertainties, along with attention to (more conventional) physiological, social and psychological health parameters, and ethical reflections on animals, clients and organizational structures. A multi-dimensional framework is therefore required to acknowledge and handle moral distress when working with animals and clients. The veterinary community, institutional structures and society should embrace the presence of existential concerns and moral distress among veterinarians and encourage moral agency as an expression of care for colleagues, animal patients and clients.
